# Frequent *NPM1* mutation, monoblastic/monocytic origin and prognostic significance of organ and system involvement in myeloid sarcoma: a multicenter study

**DOI:** 10.1002/2056-4538.70079

**Published:** 2026-03-04

**Authors:** Alex Jenei, Béla Kajtár, Tamás László, Hazem A Juratli, Livia Vida, Ágota Szepesi, Réka Mózes, Botond Timár, Jörg Halter, Stefan Dirnhofer, Alexandar Tzankov

**Affiliations:** ^1^ Department of Pathology and Experimental Cancer Research Semmelweis University, Faculty of Medicine Budapest Hungary; ^2^ Department of Pathology University of Pécs Medical School, Clinical Centre Pécs Hungary; ^3^ Histological Diagnostics Kempf and Pfaltz Zurich Switzerland; ^4^ Ekvimed Healthcare Service Ltd. Budapest Hungary; ^5^ Department of Hematology University Hospital Basel Basel Switzerland; ^6^ Institute of Medical Genetics and Pathology University Hospital Basel Basel Switzerland

**Keywords:** myeloid sarcoma, leukemia cutis, extramedullary myeloid proliferation, histiocytosis, monocyte, NPM1

## Abstract

Myeloid sarcoma (MS) is a tumorous extramedullary proliferation of blast or blast equivalent cells (e.g., promonocytes or promyelocytes). The most frequent cutaneous presentation is often referred to as leukemia cutis (LC). These lesions, especially without the clinical context of a known bone marrow disease, pose a differential diagnostic challenge. In this retrospective multicenter clinico‐pathological study on 154 patients with MS or LC, 169 samples were analyzed by morphology, immunohistochemistry, and fluorescent *in situ* hybridization, and a subset by additional sequencing [*TP53*]. The majority of cases were lysozyme positive (diffuse in 91% and focal in 5%), 51% showed diffuse and 6% focal expression of CD56, and IRF8 was strongly positive in 31% of the lesions. Lack of myeloperoxidase (MPO), CD117, and CD34 expression was observed in 27%, 39%, and 58%, respectively. PU.1 was positive in almost all instances (95%), but BRAF V600E was consistently negative. CD123 was diffusely (13%) or focally (25%) positive, which, in addition to frequent CD4 (73%) and CD56 expression, pointed to a phenotypic overlap with blastic plasmacytoid dendritic cell neoplasms. Survival analysis revealed that MS occurring at sanctuary sites (CNS, orbit, ovary, and testis) was characterized by excellent survival. Similarly to histiocytoses, there was a prognostic difference between isolated and multisystemic involvement by MS. Patients who underwent allogeneic hematopoietic stem cell transplantation showed significantly improved survival. In conclusion, this multicenter study suggests that most MS are of myelomonocytic/monoblastic origin, a high proportion of them are *NPM1* mutated, and may lack expression of MPO and CD34. NPM1 mutation‐specific antibodies should be integrated into the diagnostic panels for MS or LC, while IRF8 and PU.1 are not recommended as they cannot distinguish MS from histiocytic neoplasms.

## Introduction

Extramedullary (EM) presentations of myeloid diseases are rare. Besides some recently described emerging and sometimes conflicting entities that represent the more mature spectrum of EM myeloid tumors, for example, myelodysplasia cutis [1] and sclerosing extramedullary hematopoietic tumor (SEMHT) [2], the most frequent EM manifestation is the proliferation of myeloid blasts or blast equivalents (immature precursors, such as promonocytes in monocytic leukemias, NPM1‐positive myeloid precursors in *NPM1*‐mutated AML or the promyelocytes in acute promyelocytic leukemia with *PML::RARA* fusion), referred to as myeloid sarcoma (MS) by both the recent WHO and ICC classifications [3, 4]. However, the nomenclature of immature EM myeloid tumors is still not completely consistent: while the historical names like chloroma and granulocytic sarcoma are no longer used, some authors have raised reasonable questions regarding the term MS as well and propose the use of extramedullary acute myeloid leukemia (AML) instead [5].

The reported incidence of MS is highly variable [6, 7, 8], which is mainly due to inconsistent use of terminology; it is similar in the pediatric and adult age groups [9] and shows a male predominance [10]. MS is repeatedly underdiagnosed [6, 11, 12] and represents a major source of misdiagnoses. The most involved sites are the skin, also referred to as leukemia cutis (LC), lymph nodes, and gastrointestinal tract, but virtually any anatomical site can be affected. Agreement on which organ involvements should be considered as MS is lacking. Most studies, including the present work, do not consider spleen and liver (being hematopoietic organs) involvement as MS (except when there is a mass lesion detectable), and some authors go further and do not accept lymph node infiltration as MS either [5, 10].

Investigations into the pathogenesis of MS have implicated the potential role of several factors, such as the adhesion molecule CD56, the surface protein CD11b, the chemokine receptors CCR2, CCR5, CXCR4, CXCR7, and the complement factor C1Q, but the background of EM propagation is still not fully understood [5, 13, 14, 15]. The most common cytogenetic alterations in MS are *RUNX1::RUNX1T1* and *CBFB::MYH11* fusions, and, especially in LC. numerical abnormalities of chromosome 8 [16, 17]. In addition to the *NPM1* mutation, which is slightly more common in MS than in AML [18], RAS pathway abnormalities are significantly more common in the former [19, 20]. A recent study by Nann *et al* identified frequent alterations in the MAPK/ERK pathway, as well as *NPM1* and *EZH2* mutations [21]. Other frequent mutations include *FLT3*‐ITD and TKD, *TET2, DNMT3A*, and *IDH2* [16, 17, 22].

Because of the potential therapeutic consequences, the screening for rearrangements of *PDGFRA, PDGFRB, FGFR1, FLT3*, and *JAK2*, or *ETV6::ABL1* fusion in MS is crucial to exclude an underlying myeloid/lymphoid neoplasm with eosinophilia and tyrosine kinase gene fusions (MLN‐Eo with TKF). This applies especially to *NPM1* wild type cases, as *NPM1* mutations characteristically define a distinct molecular cluster of AML and are uncommon in cases harboring the canonical eosinophilia‐associated tyrosine‐kinase fusions, which is underpinned by the practical mutual exclusivity seen in reported cohorts [23, 24]. Of note, the EM presentation of MLN‐Eo with TKFs is common [25, 26, 27] and their general low incidence, variegated presentation, and the occasional lack of eosinophilia can make the proper diagnosis challenging.

In most instances MS presents synchronously with intramedullary AML or, commonly, emerges as extramedullary relapse following chemotherapy or allogeneic hematopoietic stem cell transplantation (HSCT). The latter is characterized by a wide anatomical distribution with a more common presentation at immunological sanctuary sites (testes, ovaries, CNS) [28, 29]. It is suggested that while graft‐versus‐leukemia (GVL) effect – regulated by alloreactive T cells via PD‐L1‐PD‐1 and CD80 interaction – enables remission in the bone marrow, it shows decreased effectiveness at EM sites [30, 31]. MS can also develop as a transformation of myeloproliferative neoplasms (MPN), myelodysplastic syndromes/neoplasms (MDS) or overlap myelodysplastic/myeloproliferative neoplasms (MDS/MPN) [32]. Isolated MS without intramedullary disease is one of the rarest clinical scenarios raising diagnostic problems [33].

As with nomenclature, prevalence and pathogenesis, there are discrepant findings on prognosis. While some authors found that MS is associated with shorter survival compared to AML [9], many could not detect significant differences between the two [34]. Conflictingly, even better event‐free‐ and overall survival has been documented too [35].

Thus, MS are diagnostically (and therapeutically) challenging entities for which, despite increasing literature data, only a limited number of comprehensive studies are available, and many questions remain to be answered. The goal of this multicentric study was to analyze a large series of MS focusing on morphological, clinicopathological, and differential diagnostic aspects. Since most of the diagnostic problems arise from the variegated immunophenotype of MS, we aimed, besides the re‐evaluation of the markers used for original diagnosis, at applying markers that have not been evaluated previously on a larger series of MS.

## Materials and methods

A multi‐center retrospective study was designed. A total of 169 samples from 154 patients (94 from the Semmelweis University Department of Pathology and Experimental Cancer Research in Budapest, Hungary; 43 from the Institute of Medical Genetics and Pathology of the University Hospital Basel, Switzerland; and 17 from the Department of Pathology, University of Pécs, Faculty of Medicine, Pécs, Hungary), diagnosed between 2010 and 2024, re‐classified according to the WHO 5 and ICC 2022 criteria were enrolled in this study. All Swiss patients have given general informed consent for their archival tissue to be used for scientific research, and in accordance with the Swiss Federal Act on Research involving Human Beings, article 38, postulating that small quantities of (such archived) bodily substances from generally consented patients are allowed to be used for irreversibly anonymized research purposes. The procedure, in accordance with the Declaration of Helsinki, was approved as such by the ethics committee of Northwestern and Central Switzerland (EKNZ 2023‐00907). All Hungarian patients gave informed consent for the research use of archival tissue material and clinical data. The study was conducted in accordance with the Declaration of Helsinki and approved by the Ethics Committee of the Hungarian Medical Research Council (BM/29372‐3/2024).

A database was constructed containing the exact immunophenotype assessed at the time of diagnosis, cytogenetic and molecular findings of the MS and, in cases in which bone marrow samples of patients were available, the diagnosis, phenotype, cytogenetic and molecular findings of the intramedullary disease as well as relevant clinical data such as sex and age, the diagnosis date and site of MS, the therapy applied, the date of last presentation and/or death, and the cause of death.

Forty‐four percent of the samples had been sent to the participating departments from external institutions for a second opinion. Most of these could not, or could only partially be used for additional immunohistochemical or cytogenetic testing, since there was very limited tissue left over after diagnostic panels had been performed. Very small samples had to be excluded as per ethics committee regulations to avoid the entire diagnostic material being used up.

Every sample was reviewed by two expert hematopathologists (AT and AJ). An immunohistochemical panel of 10 antibodies was applied, depending on the amount of material available (mutated NPM1 *n* = 71; IRF8 *n* = 54; CD123 *n* = 53; p53 *n* = 50; lysozyme *n* = 52; CD56 *n* = 51; BRAFV600E *n* = 46; PU.1 *n* = 44; PDL1 *n* = 43; PD1 *n* = 42). The panel contained antibodies related to the monocytic/histiocytic lineage (lysozyme, IRF8, PU.1), proteins playing a role in homing and immune regulation and evasion (CD56, PD1 and PDL1), and mutation specific or mutation‐suggesting antibodies (BRAF V600E, p53 and mutational‐specific NPM1) (Table 1). Either whole slides or, for a subset of cases, tissue microarrays (TMA) were used for IHC staining; TMA (6 × 9 cores, diameter 2 mm) with double cores per tissue block were constructed using a TMA Master (3DHISTECH, Budapest, Hungary). In addition, a FISH panel (break‐apart probes for *PDGFRA, PDGFRB, FGFR1, JAK2*, double‐color probes for *TP53/CEN17*, double‐fusion probes for *RUNX1::RUNX1T1, CBFB::MYH11, GATA2::MECOM, BCR::ABL1*) was applied to 24 cases (Table 1).

**Table 1:** List of the immunohistochemical markers and fluorescent *in situ* hybridization (FISH) probes applied

Antigen/Locus	Clone/Probe	Source	Dilution	Retrieval	Incubation
BRAF V600E	VE1	Abcam ab228461	1:100	CC1 64 min	12 min
CD56	MRQ‐42	Ventana 760‐4596	RTU	CC1 56 min	24 min
CD123	BR4MS	Leica NCL‐L‐CD123	1:20	CC1 40 min	32 min
IRF8	EPR20441	Abcam ab207418	1:400	CC1 40 min	32 min
Lysozyme	Rabbit polyclonal	Ventana 760‐2656	RTU	CC1 8 min	16 min
mut NPM1	Rabbit polyclonal	Invitrogen PA1‐46356	1:800	CC1 32 min	24 min
PD1	Clone NAT105	Ventana 760‐4895	RTU	CC1 48 min	12 min
PDL1	E1L3N	Cell signaling 13684	1:50	CC1 64 min	32 min
PU.1	EPR 3158Y	Abcam ab108299	1:50	CC1 16 min	48 min
*BCR::ABL1*	22q11.2/BCR_9q34.1/ABL1	Metasystems D‐5052‐100‐OG	RTU	–	Overnight
*CBFB::MYH11*	16q22/CBFB_16p13.1/MYH11	Metasystems D‐5126‐100‐OG	RTU	–	Overnight
*FGFR1* break apart	8p11.2_FGFR1	Metasystems D‐5041‐100‐OG	RTU	–	Overnight
*GATA2::MECOM*	3q21/GATA2_3q26.2/MECOM	Metasystems D‐5124‐100‐OG	RTU	–	Overnight
*JAK2* break apart	9p24_JAK2	Metasystems D‐5098‐100‐OG	RTU	–	Overnight
*PDGFRA/CHIC2/FIP1L1*	4q12_PDGFRA/CHIC2/FIP1l1	Metasystems D‐5063‐100‐TC	RTU	–	Overnight
*PDGFRB* break apart	5q32‐q33_PDGFRB	Abbott ABT6N2410	Abbott dilution protocol	–	Overnight
*RUNX1::RUNX1T1*	8q21.3/RUNX1_21q22RUNX1T1	Abbott ABT08L70‐20	Abbott dilution protocol	–	Overnight
*TP53/CEN17*	17p13_TP53	Metasystems D‐5103‐100‐OG	RTU	–	Overnight

RTU, ready to use; CC1, cell conditioning 1 solution. Abbott dilution protocol: 7 μl hybridization buffer, 2 μl distilled water, 1 μl probe.

In cases with pathologic p53 positivity, *TP53* sequencing had been conducted as previously described [36, 37]. If present in the medical records available, somatic genetic data obtained by the accredited diagnostic platforms that were active at the contributing institutions at the time of initial diagnosis were extracted and used for the purposes of the study.

Overall survival (OS) was calculated from the time of diagnosis to death or last follow‐up. Kaplan–Meier survival curves were generated and evaluated using the log‐rank test to compare survival times between groups, using IBM SPSS Statistics (version 29.0.1.0). A Cox multivariable analysis was conducted for variables of prognostic significance in the univariable analysis. The *p* values of 0.05 or lower were considered statistically significant.

## Results

### Extramedullary disease presentation

**Figure 1:**
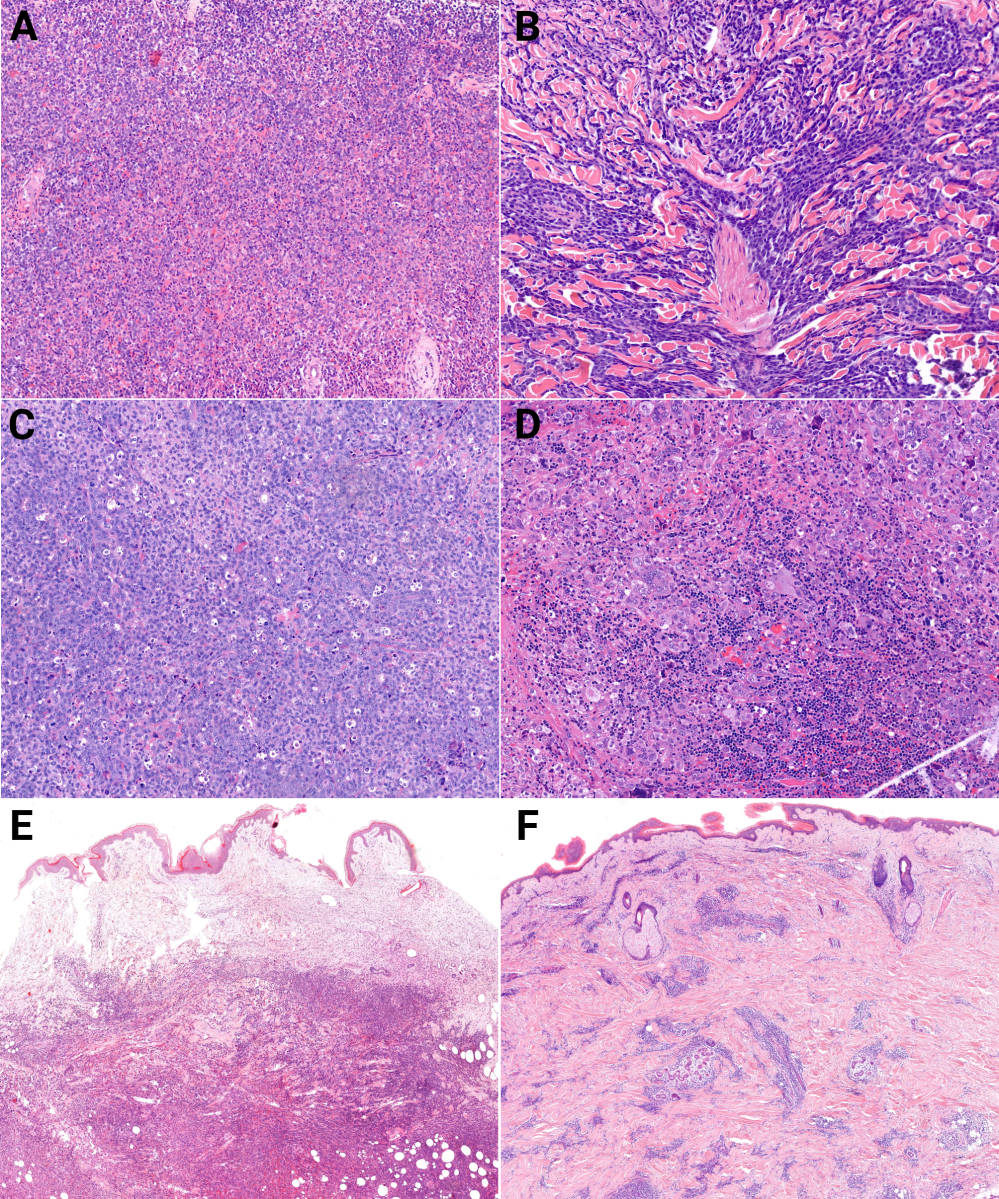
H&E features of various MS (A–D; ×20) and cutaneous (E, F; ×4) infiltration patterns. (A) MS with eosinophilia and maturation; (B) monocytic MS; (C) monoblastic MS with starry‐sky pattern; (D) erythroblastic sarcoma with marked pleomorphism; (E) diffuse, dense, destructive infiltration – cutaneous MS pattern; (F) perivascular and periadnexal infiltration *–* leukemia cutis pattern.

The MS in our series that lacked significant ‘crush’‐artifacts most often (60%) presented as monotonous infiltrates of medium (10–20 μm) to large (>20 μm) immature blasts with abundant basophilic cytoplasm, irregular nuclei, and prominent nucleoli. A ‘starry‐sky’ appearance due to prominent apoptotic activity could be observed in 12% of cases. A subset of cases (19%) consisted of medium sized monocytic cells with folded nuclei without prominent nucleoli. 7% of the MS displayed a degree of maturation and consisted of EM hematopoiesis‐like infiltrates intermingled with a blast population >20%. Rarely (4%) tumors were composed of pleomorphic cells reminiscent of large‐cell lymphomas or even non‐hematopoietic tumors (Figure 1).

Of all the organs examined histologically, skin involvement was the most common (Figure 1). The infiltration of variably dense immature monocytoid or blastoid cells was exclusively dermal without epidermotropism, characteristically with perivascular and/or periadnexal arrangement. Based on the density of the infiltration and its interference with the tissue architecture, we distinguished between tissue‐sparing, so‐called LC (49%) and destructive, dense, tumorous cutaneous MS patterns (51%), which both showed no difference in clinical behavior (data not shown). Lymph node involvement, as the second most frequent location, presented as interfollicular, diffuse, or, rarely, sinusoidal and/or subcapsular infiltration.

The reviewed IHC stains used for the original diagnosis represented a broad spectrum of antibodies. The panel used was highly dependent on whether intramedullary disease was known before the MS diagnosis or not. Since 21 cases preceded the detection of BM disease and 13 MS turned out to be isolated forms, the initial work‐up of these 34 instances was laborious.

The results for the most frequently used diagnostic antibodies and the designed panel markers are summarized in Table 2.

**Table 2:** Immunophenotype of MS

Marker groups	Antigen	Positive		Partial/dim		Negative	
**Myeloid and immaturity markers**	**MPO**	65/135	48%	34/135	25%	36/135	27%
	**CD117**	42/98	43%	18/98	18%	38/98	39%
	**CD34**	33/122	27%	18/122	15%	71/122	58%
**Monocytic/histiocytic markers**	**lysozyme**	100/110	91%	6/110	5%	4/110	4%
	**IRF8**	17/54	31%	11/54	20%	26/54	48%
	**PU.1**	42/44	95%	Ø	Ø	2/43	5%
**Mutation‐specific/related**	**NPM1 mut**	28/71	39%	Ø	Ø	43/71	61%
	**p53** *	10/50	20%	37/50	74%	3/50	6%
	**BRAF V600E**	0/46	0%	Ø	Ø	46/46	100%
**Immune regulation and evasion**	**CD56**	34/67	51%	4/67	6%	29/67	43%
	**PD1**	0/42	0%	Ø	Ø	42/42	100%
	**PDL1**	5/43	12%	Ø	Ø	38/43	88%
**Miscellaneous**	**CD123**	7/53	13%	13/53	25%	33/53	62%

The results for the most frequently used diagnostic antibodies and the designed panel markers (the panel is performed by the Institute of Medical Genetics and Pathology of the University Hospital Basel, Switzerland). Partial refers to the staining pattern, meaning partial positivity, while dim refers to weak staining. Color codes: the different shades (1–30% light, 31–70% medium, 71–100% dark) correlating with the higher percentages.

* p53 positive = strong expression, suggestive of mutation; partial/dim = wild‐type positive; negative = zero phenotype, suggestive of mutation.

Monoblastic‐monocytic/myelomonocytic differentiation could be observed in the overwhelming majority of cases and was characterized by the expression of lysozyme (diffuse in 91% and focal in 5% of the cases), often in combination with IRF8 (31% diffuse and 20% partial positive). As expected [38], a sizable proportion of cases (51% diffuse and 6% focal) expressed the adhesion molecule CD56. MS with erythroid differentiation, supported by the co‐expression of CD71, glycophorin A and E‐cadherin, and LMO2, applied to three cases, while megakaryoblastic differentiation, supported by the positivity of the blasts for CD42b, CD61, osteonectin and factor VIII, to two cases.

Importantly, the initial diagnosis of samples submitted for consultation was often lymphoma of either B‐ or T‐cell lineage based on the expression of various B‐ and T‐cell lineage markers. Indeed, CD79a expression was detected in three cases, all of them being focal. One of them was a *CBFB::MYH11* rearranged MS, while the others co‐expressed PAX5 and one harbored *RUNX1::RUNX1T1* fusion, which can be observed in respective instances [39, 40]. In the setting of extramedullary monoblastic/monocytic lesions, CD4 was not surprisingly commonly expressed (22 positive out of the examined 30 cases), which can be a source of misdiagnosis on the basis of phenotypic overlap with blastic plasmacytoid dendritic cell neoplasms (BPDCN) (especially if CD56 and/or CD123 is co‐expressed; see below), as well as T‐cell lymphomas. Six MS displayed CD7 and at least five cases showed focal CD30 positivity. Unlike in most histiocytes, the proliferation rate (Ki67) was uniformly high, ranging between 50% and 90%. The histopathological features, including complete phenotype data of all samples, are available as supplementary material, Table S1.

**Figure 2:**
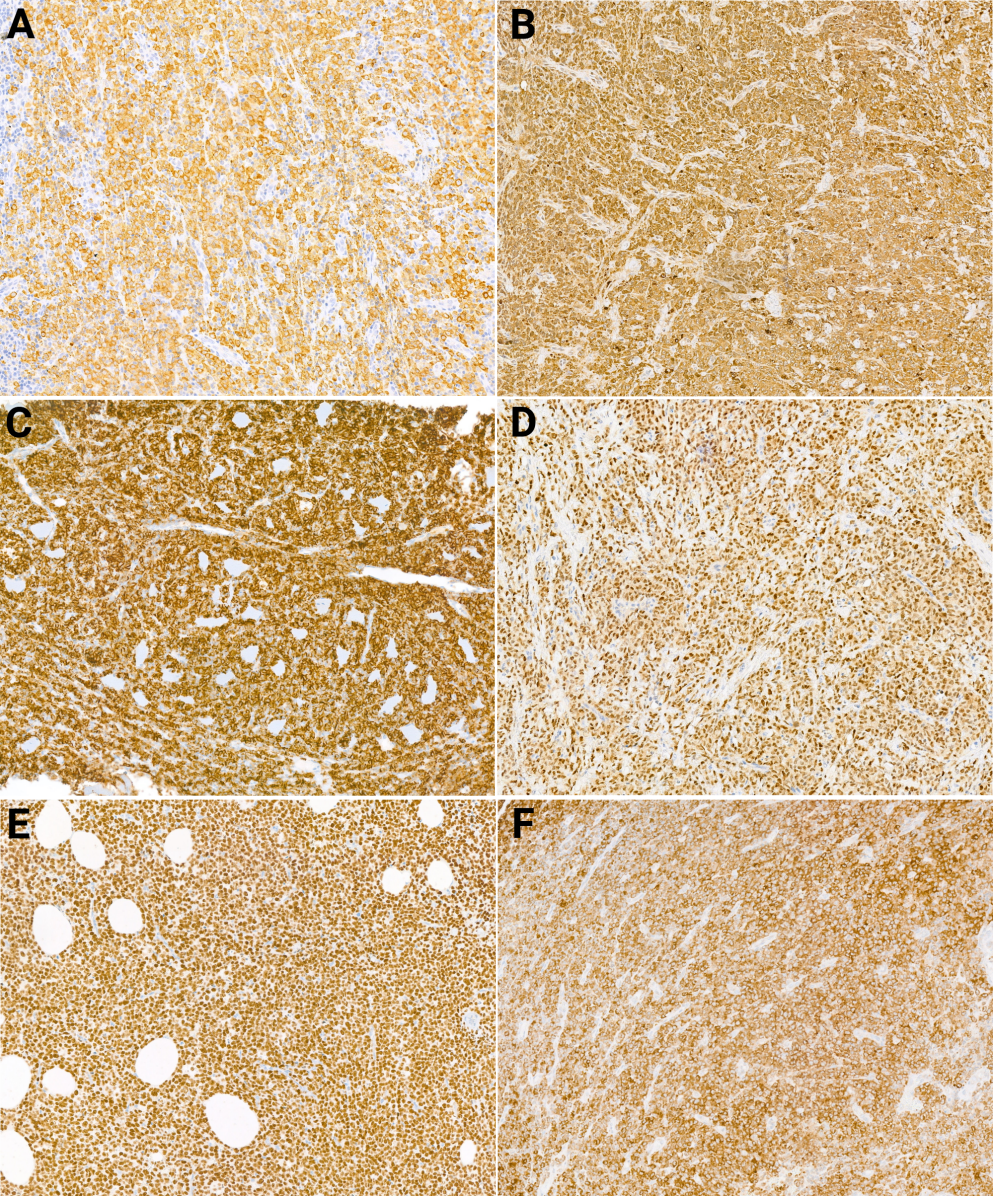
Immunohistochemical characteristics of MS. (A) MPO positive myeloid blasts; (B) lysozyme positive monoblasts; (C) diffuse CD56 expression; (D) IRF8 positive MS; (E) diffuse PU.1 expression; (F) uniform, strong cytoplasmic expression of mutated NPM1.

Mutated NPM1 positivity detected by immunohistochemistry was present in 39% of cases. IRF8 was diffusely nuclear positive in 31% of the instances. PU.1 was positive in almost all cases (95%), but none turned out to be BRAF V600E positive. CD123 was diffusely (13%) or focally (25%) positive in a non‐negligible portion of the cases; the staining was typically moderate and only occasionally strong. 80% of these CD123 positive tumors also expressed CD56, and a half of the tested cases were CD4 positive as well, highlighting further phenotypic overlaps between MS and BPDCN. Mutation‐suggesting pattern of p53 expression was present in 13 cases (10 strong positive and 3 completely negative). PDL1 was expressed by five tumors; surprisingly, none of them homing to immune‐privileged sites (lymph node *n* = 3, skin *n* = 1, tibia *n* = 1), while PD1 was negative in all tested cases, and there were no significant amounts of PD1^+^ tumor‐infiltrating lymphocytes (Figure 2).

### Cytogenetic and molecular findings

**Figure 3:**
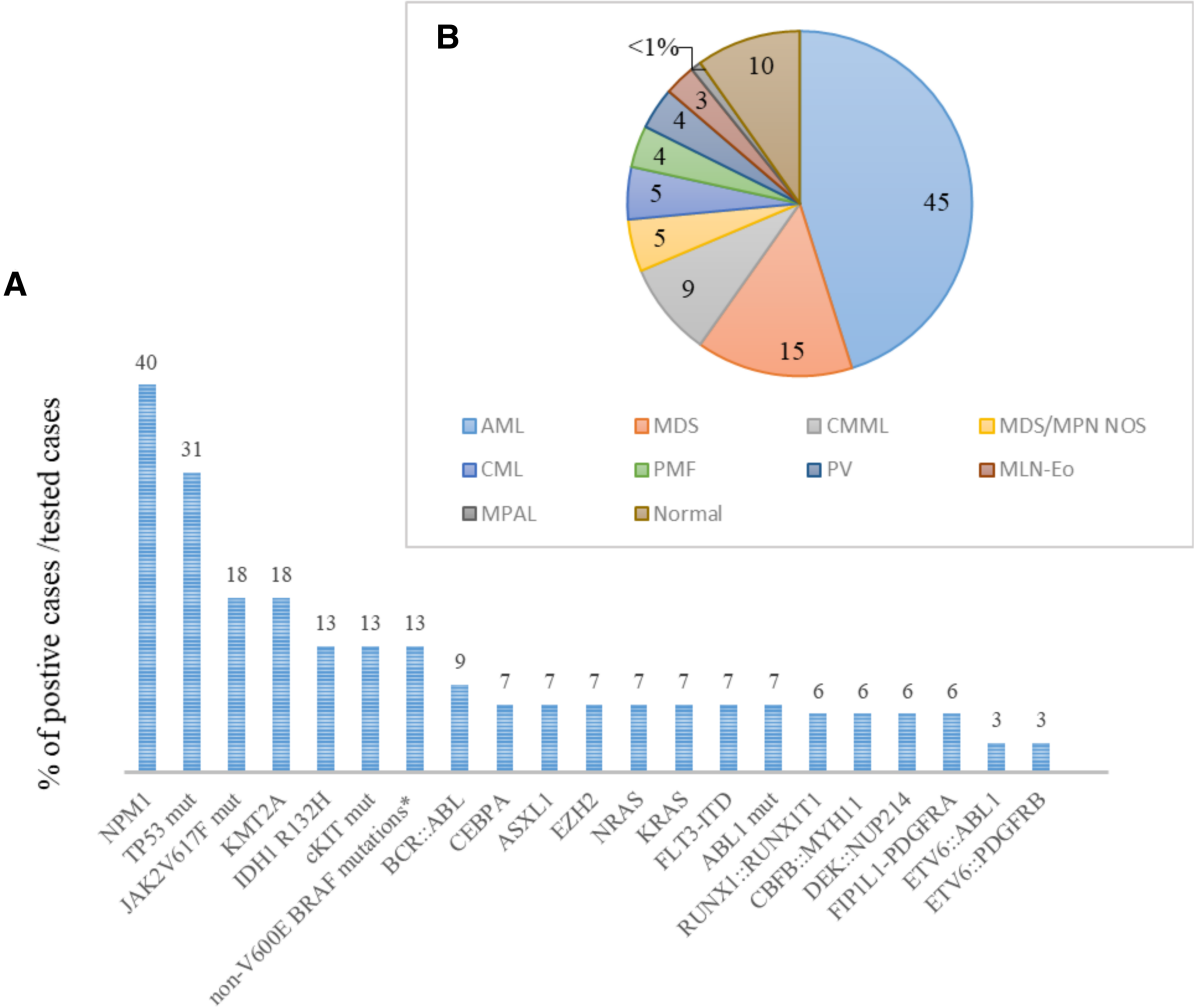
(A) Cytogenetic and molecular features of MS. The chart shows the percentage of positive cases among those evaluated for the given genetic alteration. *NPM1 n* = 32/81; *TP53 n* = 5/16; *JAK2V617F n* = 3/17; *KMT2A n* = 3/17; *c‐KIT n* = 2/15; *IDH1R132H n* = 2/16; *CEBPA n* = 1/14; *AXSL1 n* = 1/14; *BCR::ABL n* = 3/32; *RUNX1::RUNX1T1 n* = 2/32; *CBFB::MYH11 n* = 2/32; *DEK::NUP214 n* = 2/32; *FIP1L1::PDGFRA n* = 2/32; *ETV6::ABL1 n* = 1/32; *COL1A1::PDGFRB n* = 1/32. (B) The percentage of bone marrow findings in 133 MS patients.

In addition to the cases stained for mutated NPM1, eight cases were reported positive by sequencing; thus, altogether *NPM1* mutation was detected in 40% of tested cases, making it the most frequent alteration in this cohort. In seven cases of our cohort, for which both mutational and IHC data were available (*n* = 7), there was a 100% correlation of the obtained results. *TP53* mutation was additionally confirmed in five cases. Interestingly, for four cases, there was IHC and *TP53* mutational data, of which only two of the cases with p53 IHC staining patterns suggestive of mutations turned out harboring a mutation, questioning the robustness of IHC to predict mutations in hematolymphoid tumors [41]. A *FIP1L1::PDGFRA* fusion was detected in two cases (6% of all tested cases), while *COL1A1::PDGFRB* and *PCM1::JAK2* in one case each (3%). None of the tumors were *BRAF* V600E mutated but non‐V600E mutations were present in two tumors: one MS with *BRAF* G469A and another with *BRAF* K601E mutation. The case harboring G469A showed co‐mutations of *TP53*, *EZH2*, and *NRAS* while the K601E mutated MS was *NPM1* co‐mutated. These findings are in line with other observations that advanced AML can gain *BRAF* mutations [42]. Further molecular findings extracted from the medical records of diagnostic tests performed on FFPE biopsies of the MS at the time of original diagnosis are shown in (Figure 3).

### Bone marrow findings

Information regarding the bone marrow was available for 133 patients. Thirteen patients had isolated MS with normocellular bone marrows lacking any atypical features. Among bone marrow diseases, AML was the most common finding (*n* = 61).

In the background of extramedullary transformation, MDS was the most common finding (*n* = 20), followed by CMML (*n* = 12), CML (*n* = 6), MDS/MPN NOS (*n* = 6) and so‐called Philadelphia negative MPN (PV in five cases, PMF in five cases). MLN‐Eo with TKF was diagnosed in four cases (*FIP1L1::PDGFRA* in two cases, *COL1A1::PDGFRB* in one and *PCM1::JAK2* in one); mixed‐phenotype (B/myeloid) acute leukemia in one patient.

### Comparative findings

In comparison to their intramedullary disease counterparts, 64% of the non‐isolated MS cases showed some phenotypic deviation of the neoplastic population from the bone marrow blasts in one or more markers. Loss of CD34 expression was the most frequent deviation (55%), followed by gain of CD56 (31%) and loss of MPO (10%).

No discrepancies were observed in the cases in which comparative FISH testing of the MS and intramedullary myeloid neoplasia was available, but the number of such cases was low (*n* = 11).

Comparable information regarding molecular genetic alterations for both bone marrow and extramedullary samples was available for 28 patients. In the vast majority of cases (*n* = 25), the findings from the diagnostic panel performed on the bone marrow samples were confirmed by targeted mutation analysis (or, in the case of NPM1, by immunohistochemical testing) on the MS samples. No discrepancies were observable for *NPM1*, *TP53*, *BRAF* K601E, *ABL1, IDH1* and *KIT* mutated intramedullary myeloid neoplasia and the respective MS pair. In one exceptional case of an extramedullary transformation of a *JAK2* V617F positive PMF, the MS turned out to be NPM1 positive by IHC and *JAK2 V617F* wild‐type, consistent with known negativity for driver mutations in more than 50% of blast phase MPN [43].

Large NGS panels run on the intramedullary myeloid neoplasia and MS applied to only four instances. No discrepancies could be detected between an AML with *DEK::NUP214* fusion and *FLT3‐*ITD mutation, an *NPM1* and *IDH1* R132H co‐mutated AML and an *NPM1* mutated AML with 8q trisomy and the respective MS pair. However, in an *NPM1* and *FLT3‐*ITD co‐mutated AML, in the MS, which presented as an extramedullary relapse after transplantation, *NPM1* was mutated, but *FLT3*‐ITD could not be detected, in line with the proposed secondary nature of *FLT3* alterations in *NPM1* mutated AML [44].

### Clinical features and outcomes

**Figure 4:**
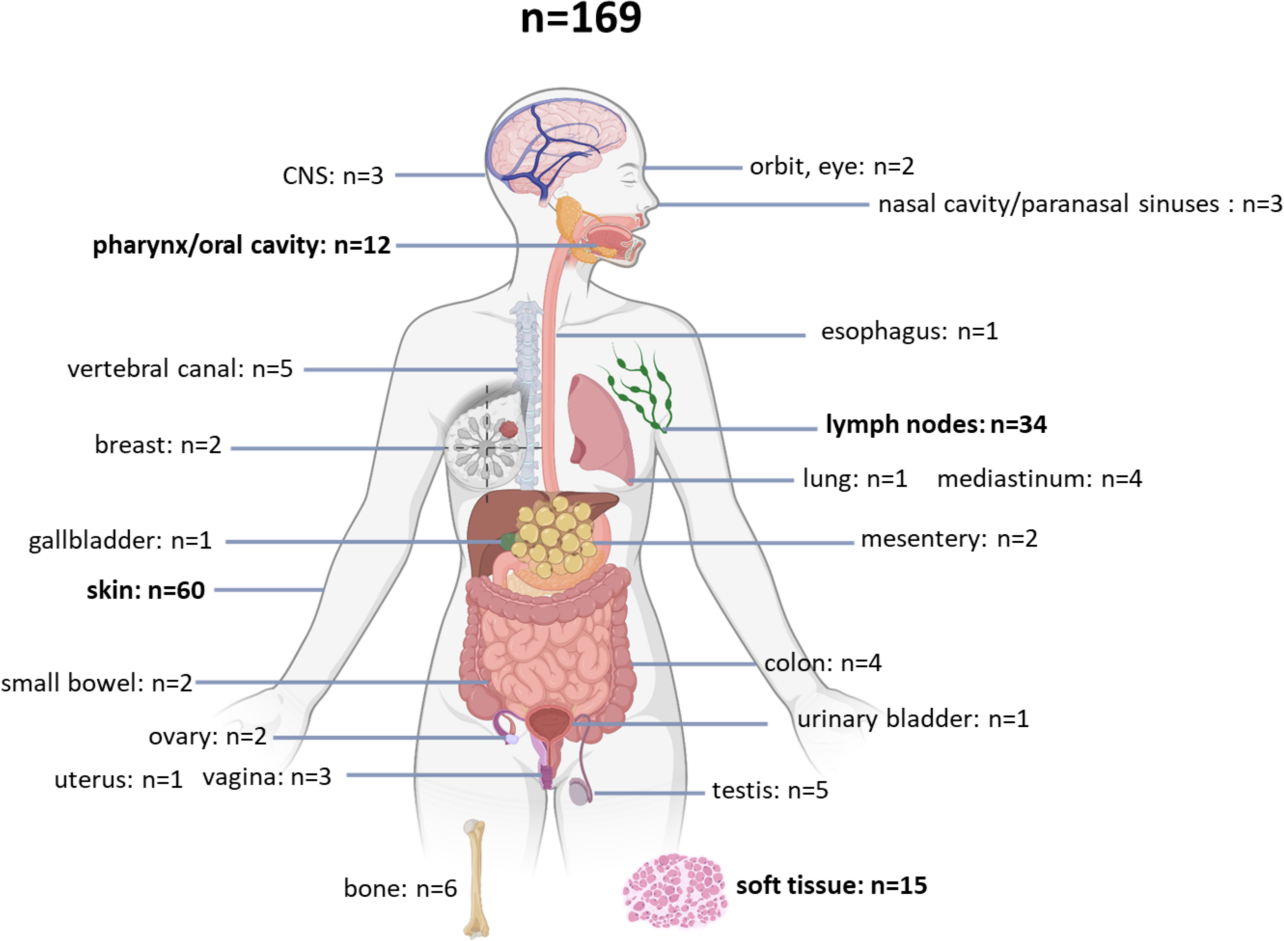
Anatomical distribution of 169 MS samples from 154 patients. The most common locations are marked in bold. Created with BioRender.com.

The average age of the patients at diagnosis was 55 years, there was a male predominance with a female‐to‐male ratio of 1:1.4. Almost all anatomical sites were affected (Figure 4), but the most common location was the skin (36%), followed by lymph nodes (20%), soft tissues (9%), and the oral cavity (7%), respectively.

Clinically, skin lesions were usually detected as multiple nodules, plaques, or papules. Lymph node involvement was recognized in the form of solitary or multiple painless lymphadenopathies. MS affecting other organs presented as mass‐forming tumors.

As mentioned above, MS were most often diagnosed as an extramedullary infiltration in the background of pre‐existing or synchronously detected AML (*n* = 61), but also frequently (*n* = 53) represented the EM transformation of chronic myeloid neoplasms (CMN). EM relapses after HSCT applied to 19 instances. In 13 cases, MS was isolated, without evidence of bone marrow involvement.

**Figure 5:**
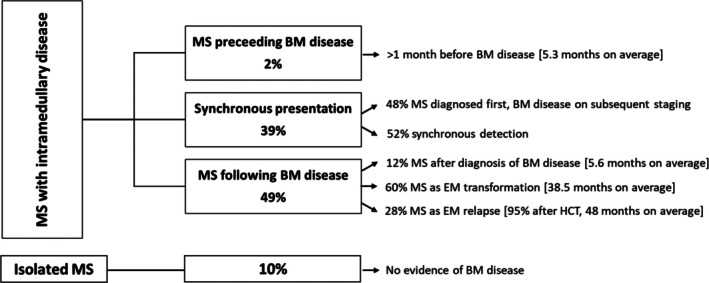
Temporal relationship between the onset of the MS and bone marrow disease.

The temporal relationship between the onset of the MS and bone marrow disease is shown in Figure 5.

Information regarding treatment modalities was available for 95 patients, 39 of whom underwent HSCT. The transplantation rate was 75% for the Swiss and 25% for the Hungarian cohort. Clinical characteristics of the 154 patients with MS involved in the study are presented in supplementary material, Table S2.

**Figure 6:**
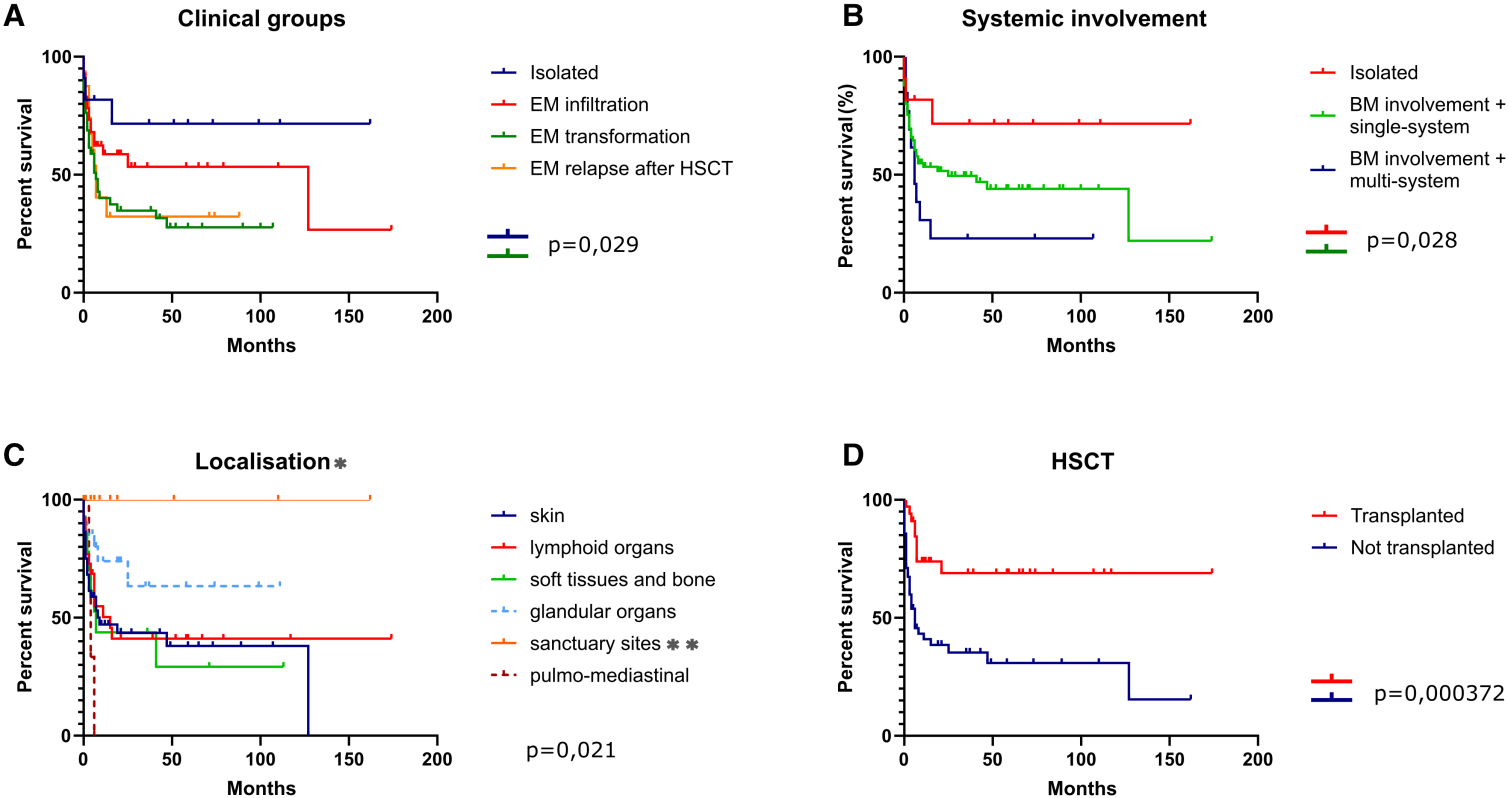
Overall survival of MS patients according to (A) clinical groups, (B) systemic involvement, (C) anatomical location and (D) transplant state. *To facilitate the examination of the extremely diverse locations, we have grouped the various organ involvements into categories. Skin: cutaneous infiltration. Lymphoid organs: lymph nodes and tonsils. Bone and soft tissue: as the name indicates. Sanctuary sites and orbit: CNS, testis, ovary. **Orbit is traditionally not considered as an immune‐privileged site *sensu stricto*, yet our decision to include the two cases of orbital MS into this category is based on the susceptibility of this tissue to a plethora of specific immune‐mediated disorders such as Graves' orbitopathy, IgG4‐related orbital disease and other pseudotumors, suggesting that some beyond‐barrier properties may apply to it. Pulmo‐medisatinal: lung and mediastinum. Glandular organs: we have classified organs containing either exocrine or endocrine glandular tissue as glandular organs, such as salivary glands, breast, oral and nasal cavity, gastrointestinal organs, and female genital organs, with the exclusion of ovary.

Survival data was available for 122 patients. Fifty‐three patients died of disease. The median OS was 19 months. Kaplan Meier overall survival curves for assumingly meaningful clinical groups (EM disease in the background of AML/EM transformation in the background of CMN/EM relapse after HSCT/isolated EM disease) are shown in Figure 6A, the comparison of single‐ and multisystem involvement by MS in Figure 6B, the impact of HSCT in Figure 6C and OS according to the anatomic location in Figure 6D. Among these variables, the anatomic location (*p* = 0.021) and HSCT (*p* = 0.000372) appeared to have a significant prognostic value on outcome. Furthermore, comparing clinical groups, significant differences were detected between isolated occurrences and extramedullary transformation (*p* = 0.029), and between isolated and multisystemic involvement (*p* = 0.028). However, there were no survival differences regarding NPM1 mutation status or tumorous versus LC presentation of skin lesions. Multivariable analysis showed that HSCT can meaningfully decrease the risk of mortality (relative risk 0.239, 95% CI 0.112–0.510, *p* = 0.000213), while multifocal and especially multisystem involvement is linked to dismal outcomes (relative risk 2.458, 95% CI 1.375–4.452, *p* = 0.003). All other factors did not reach statistical significance.

## Discussion

This large multi‐center study corroborates known facts and considerably expands current knowledge on the clinical impact and histopathologic presentation of MS.

We iterate that while the diagnosis of MS in the clinical context of a known myeloid disease is usually straightforward, significant phenotype changes of the blasts may occur making an accurate diagnosis difficult. Although gain of CD56 expression at EM sites has been documented [45], a particular finding with important diagnostic consideration is the frequent lack of CD34 in 58% and lack of MPO in 27% of cases.

Recognition of MS outside of the context of a known myeloid disease can be challenging, particularly bearing in mind the possibility of dealing with double negativity for MPO and CD34. This is relevant as, in our large series, both isolated MS and cases in which the MS was detected before intramedullary disease were responsible for 22% of all instances. With respect to diagnostics, our data emphasize the high utility of lysozyme, PU.1, IRF8, and especially mutated NPM1 in recognizing MS. Lysozyme and PU.1, tested on the first occasion in a large series of MS, were positive in >90% of our cases. While lysozyme and PU.1 can be helpful in differentiating MS from other discohesive (skin) tumors, there is a caveat in that respect as histiocytic disorders (including histiocytic sarcoma) also express lysozyme and PU.1 as well as CD4 and CD68, both being also commonly expressed in MS. Convoluted nuclei, frequent skin involvement, and presence of scattered eosinophilic granulocytes are also overlapping features between histiocytic disorders and MS. For the differential diagnosis of MS from histiocytic disorders, additional markers are needed. In respectively mutated cases, BRAF V600E might be helpful, as, also corroborated by our data, V600E *BRAF* mutations are very rare to absent in MS (G466E being reported by Nann *et al* [21] and G469A and K610E being observed in our series), while affecting up to 50% of histiocytic neoplasms. Another such marker might be PDL1, which is commonly expressed in histiocytoses, while only a few MS cases (in our series – 12%) express PDL1 [46]. IRF8, originally described as a robust monoblastic, but also a plasmacytoid dendritic cell (PDC), marker [47, 48, 49], might be another helpful tool in that consideration as it was expressed in 51% of our cases, yet a recent study revealed that it can be observed in a subset of histiocytic lesions, too [50]. The most reliable marker considering the differential diagnosis MS *vs*. histiocytic neoplasms seems to be detection of mutated NPM1, applying to almost 41% of MS and 50% of LC, that precludes the diagnosis of the latter disease group [51]. CD123, though rarely (13%) diffusely positive in our MS series, is a caveat with respect to BPDCN, which may be also resolved by application of the mutational‐specific NPM1 antibody as *NPM1* mutated MS (being commonly CD123+) will stain positive, while BPDCN are per definition *NPM1* wild type [52]. Cases with at least focal CD123 expression (*n* = 20) were heterogenous. Six cases were positive for mutated NPM1. Among the NPM1 negative cases, *TP53* mutation, *KMT2A* rearrangement, *CBFB::MYH11*‐ *BCR::ABL1* fusion, and *CEBPA* mutation were detected in one–one case each. Five cases presented as an extramedullary infiltration of AML, while others the extramedullary transformation of CMML (*n* = 2), CML (*n* = 1), MDS (*n* = 1), and MDS/MPN NOS (*n* = 1).

Finally, we observed six cases with CD7 and five cases with focal CD30 positivity. While the aberrant CD7 expression on leukemic blasts is well known, applying to 23–29% of AML [53, 54], the latter is rarely reported [55, 56] and can be a huge diagnostic pitfall, especially when considering lymphomas. To sum up, expression of the mutated NPM1 protein is robust at extramedullary sites and its detection by mutation‐specific antibodies can and should be diagnostically utilized in any suspected MS [57]. Application of the V600E mutational‐specific BRAF antibody and to a part of PDL1 might be helpful in selected cases as an expression of these proteins should question the diagnosis of MS.

MLN‐Eo with TKF presented as extramedullary masses in four cases, being in line with their known propensity for EM manifestation and once again pointing toward the utmost importance of applying purposeful technologies to MS, especially to MS lacking AML‐defining genetic aberrations, to identify such cases [25, 26, 27].

Within the study, we identified two specific MS presentations, being worthy of specific discussion. Erythroblastic sarcoma, a rare form of EM presentation [58], applied to three adult cases in this cohort. All of them affected the lymph nodes and were characterized by the co‐expression of E‐cadherin, glycophorin A, LMO2, and CD71. Interestingly, one case represented an extramedullary manifestation of MLN‐Eo with *PCM1::JAK2* fusion, another was a *JAK2* V617F mutated blastic tumor in a patient suffering from PV, while the third case was an EM transformation of an MDS with *TP53* mutation. The patient with *PCM1::JAK2* is alive after 54 months of follow‐up; the patient with *TP53* mutation died of disease within weeks, while the third patient was lost from follow‐up. Two cases of megakaryoblastic sarcoma, an exceedingly rare subtype of MS [59], were present. Both cases represented EM transformation of MPN (one arising from CML and one from PV), were multisystemic, and the patients rapidly died of disease.

The prognostic factors of MS are controversial and our study on a large cohort, yet retrospective, yields important results. The median survival of our patients of 19 months was similar to the findings of other multicenter studies [26]. Also in line, HSCT significantly improved survival, without evidence of loss of this survival benefit with time, as concluded recently by Sun *et al* [60].

The wide range of anatomical locations in our cohort allowed for site‐specific survival analysis and showed that MS occurring at traditional sanctuary sites and the orbit are characterized by excellent survival, which is a particularly new finding deserving further verification and additional research with a focus on site‐specific GVL effects.

An interesting pilot study [61] introduced a new perspective on the differences between structure‐preserving infiltration, often referred to as (myeloid) LC, and mass forming skin lesions, called MS. As distinct differences in their expression profiles were identified, the authors suggested that the two should be considered as separate entities. Accordingly, we sought to see if these biological differences could be translated into divergent clinical outcomes. Our data failed to prove a survival impact on whether the skin infiltration was pure LC or cutaneous MS; indeed, by the time a skin biopsy has been performed because of clinical concerns and found positive on histologic examination, the clinical outcome was similar irrespective of the infiltration pattern, a finding which is also supported by the prognostic impact of histologically verified versus clinically suspected MS and LC in a large cohort [62].

*NPM1* mutation is considered as a favorable prognostic factor in AML [63]. However, a study by the U.S. Bone Marrow Pathology Group found that *NPM1* mutated AML with MS are genetically distinct and associated with inferior survival compared to *NPM1* mutated AML without MS [64]. These data raise the possibility that *NPM1* mutated AML with extramedullary manifestation may represent a distinct biological subgroup. Our data indirectly supports this conclusion, as we were not able to observe any survival difference between the *NPM1* mutated and unmutated MS.

Based on observations in histiocytic disorders, and their frequent clonal relationship with various BM neoplasms [65], we classified MS into groups according to their association with intramedullary disease and uni‐ or multisystemic presentation. Indeed, the resulting survival curves were reminiscent of those observed in histiocytic neoplasms (e.g., indeterminate cell histiocytosis or Langerhans cell sarcoma) [66, 67, 68, 69]. MS with intramedullary disease were associated with poorer outcomes, and multisystem involvement resulted in even worse survival rates, while isolated/unisystemic disease was linked to best outcomes. These findings propose that the clinical behavior may be independent of the phenotype, but is rather influenced by the presence of an associated bone marrow disease and/or systemic involvement.

The main limitations of our study are its retrospective nature with a non‐negligible subset of patients who were lost to follow‐up, and the restricted availability and/or eligibility of FFPE material for additional immunohistochemical and genetic studies. Also, comparative molecular analysis between MS and the intramedullary AML was possible in only a few cases. Yet, these limitations were partly counterbalanced by the size of the collection and its multicentric design.

Accounting for the limitations of the study, our results nevertheless provide evidence that isolated MS may be different from extramedullary manifestations of otherwise bone marrow‐centered myeloid diseases. This observation may impact on the terminology in the long term, namely the restriction of the use of MS to lesions that are isolated, while other lesions being better designated as extramedullary AML, or extramedullary blastic transformation (in non‐AML patients suffering from MDS or MPN).

In conclusion, through the analysis of a multicenter cohort of 154 MS we could show that most instances are of myelomonocytic‐monoblastic origin, a high proportion of them are *NPM1* mutated, there is a recurrent lack of traditionally used myeloid markers such as MPO, CD34 and CD117, and occasional aberrant expression of B‐ or T‐cell markers. While based on our findings the inclusion of NPM1 and BRAF V600E mutational‐specific antibodies into the diagnostic panels for MS is highly advisable, IRF8 and PU.1, tested on the first occasion on a large series of MS, are not recommended as they cannot distinguish MS from histiocytic neoplasms and will be expressed by a plethora of B‐cell lymphomas. Besides the phenotypic overlap with histiocytoses, MS cases show similarities in clinical outcomes when categorized analogously into uni‐ and multisystemic diseases.

## Author contributions statement

AJ conducted the investigation, analyzed the data, and drafted the manuscript. BK supplied patient samples and follow‐up data. HAJ evaluated IHC stains. TL contributed to the statistical analysis. LV, ÁS, RM, JH and SD supported clinical data collection and supplied patient samples. AT conceptualized the study, supervised the research, and partially wrote the manuscript. All authors have approved the final version of the manuscript.

## Supporting information


**Table S1.** Pathological features of all MS samples of the study

**Table S2.** Clinical characteristics of the 154 patients with MS in the study

## Data Availability

All the data generated are presented in the study. Further enquiries can be directed to the corresponding authors.
